# Investigation of antibacterial, acid and bile tolerance properties of lactobacilli isolated from Koozeh cheese

**Published:** 2012

**Authors:** Hassan Hassanzadazar, Ali Ehsani, Karim Mardani, Javad Hesari

**Affiliations:** 1 *Department of Food Hygiene and Quality Control, Faculty of Veterinary Medicine, Urmia University, Urmia, Iran; *; 2 *Department of Food Science and Technology, Faculty of Agriculture, Tabriz University, Tabriz, Iran.*

**Keywords:** Acid and bile tolerance, Lactobacillus, Probiotic, Antibacterial activity, Lactic acid bacteria

## Abstract

*Lactobacillus *strains are a major part of the probiotics, microflora of the intestine and of fermented dairy products, and are found in a variety of environments. The aim of this study was to find out the ability of bile and acid tolerance and antibacterial properties of the twenty eight isolates of three group *lactobacilli* namely *Lactobacillus plantarum*, *Lactobacillus casei* and *Lactobacillus delbruki*. For this purpose Twenty eight different *Lactobacillus *strains that isolated from Koozeh cheese as a traditional cheese were screened. The acid tolerance test was studied under pH 2.0 and 3.0 with 7.5 as control. The cell count for the acid tolerance test was obtained at an interval of 0, 1, 2 and 3 hours respectively and was pour plated on Man, Rogosa, and Sharpe (MRS) agar to be incubated at 37 °C for 24 hours. All cells were selected for bile tolerance test in MRS broth containing bile concentrations of 0% as control and 0.3% as test. Then cell counts were enumerated after 24 hours of incubation on MRS agar. Results showed twenty seven isolates did not have ability to tolerate acid and bile salts and antimicrobial activity against four indicator bacteria included *Eshirichia coli*, *Listeria monocytogenesis*, *bacillus cereus*, *Salmonella entritidis*. Only one Isolate namely *Lactobacillus casei* could tolerate acid and bile salt and had antibacterial activity against of *L. monocytogenesis*. Therefore we can consider this strain as a native probiotic but extra examinations was required.

## Introduction

Koozeh cheese is a traditional semisoft cheese produced from raw sheep milk in northwest provinces of Iran and its neighboring countries such as Iraq and Turkey. The genus *Lactobacillus *is one of the genera included in Lactic acid bacteria. All of them are Gram positive, non-spore-forming, usually catalase-negative, non-motile, and facultative anaerobic unless otherwise stated. It is also very heterogeneous, encompassing species with a large variety of phenotypic, biochemical, and physiological properties. 

*Lactobacilli* are wide spread in nature, and many species have found applications in the food industry. Lactobacilli are found where rich, carbohydrates containing substrate are available, and thus, in a variety of habitats such as mucosal membranes of humans and animals, (mainly in oral cavity, intestine, and vagina) and on plant material and fermenting food such as cheese.^1^^,^^2^ Lactobacilli are strictly fermentative, aero-tolerant to anaerobic, aciduric or acidophilic and they have complex nutritional requirements.^3^ Most stains of *lactobacillus* are classified as probiotics. 

Probiotics termed as live micro-organisms which, when evaluation administered in adequate amounts, confer a health benefit to the host.^4^ According to the guidelines of the of probiotic organisms, reported by a joint FAO/WHO working group, two of the currently most widely used *in vitro *tests are resistance to gastric acidity and bile compounds based on both survival and growth studies.^5^^,^^6^ In addition, evaluation of antagonistic effect of probiotics against various pathogens is other item for their confirmation. Further investigation may be performed to test the other abilities of probiotics such as reducing of blood cholesterol level and etc. because of the strain dependency of health promoting properties of probiotics.^7^ These evaluations are obtained through the application of one of the following methods; a) by assessing the strain’s ability in bringing about changes in optical density at the presence of various concentration of acid and bile salt^5^^,^^8^ and by evaluating growth on culture media and their antibacterial effects.^3^

 The time from entrance to release from the stomach was reported to be approximately 90 min.^9^ However, further digestive processes have longer residence times and thus, there is a need for the bacteria to be resistant to the stressful conditions of the stomach and upper intestine, which contain bile.^10^

It is necessary to find new probiotics among native strains.^5^^,^^11^^,^^12^ Traditional fermented dairy foods such as various cheeses are good reserves for the genus of *lactobacilli* as probiotics. The aim of this study was to evaluate of acid and bile tolerance and antibacterial properties of *lactobacilli* genera isolated from Koozeh cheese as a popular traditional cheese. 

## Materials and Methods

**Sampling. **Twenty four samples of koozeh cheese were purchased from ten cities of West Azerbaijan province of Iran. 

**Bacterial strains** **and culturing conditions.** Twenty eight isolates of Lactobacillus belonging to three group of *Lactobacillus* genus namely *Lactobacillus plantarum, Lactobacillus Casei and Lactobacillus delbruki* (table 1), isolated from the cheese samples that were previously identified by morphological and biochemical assay (such as carbohydrate fermentation, Arginine hydrolase, gas production of Glucose and catalase test). All of these isolates were grown in MRS broth at 35 °C for 24 hours.

**Table 1:** Name of various isolated *Lactobacillus*

***Lactobacillus*** ** genus **	**Isolate number **
***L. plantarum***	1,2,3,6,7,8,9,10,11,12,13,14,15,16,17,18,23,24,28
***L. casei***	4,5
***L. delbruki***	19,20,21,22,25,26,27

**Tolerance to acidic pH values. **Strains were grown in MRS broth at 37 °C overnight, 0.1 mL aliquots of each active cultures were adjusted to pH 3.0, 2.0 with 5 N HCl and incubated at 37 °C for 3 hours. Samples were taken every hour for 3 hours and the viable number of bacteria were enumerated by pour plate counts of all samples using 10-fold serial dilutions prepared in 0.1% peptone water.^3^ Simultaneously, bacterial growth was monitored by measuring absorbance with a spectrophotometer (Nova Spec II, Pharmacia) at 600 nm.^10^ All the experiments were replicated twice. 

**Bile Tolerance. **Strains were grown in MRS broth at 37 °C overnight; saturated bile solution was prepared separately by dissolving powdered bile extract (Oxoid). Bile solution was then filter sterilized by 4 micron filter and was added to two of the cultures to achieve a final concentration of 0.3 % and the second culture with 0 % bile served as a control sample. The cultures were incubated at 37 °C for 3 hours and then every hour for 3 hour. Viable counts of *Lactobacillus *strains were determined by pour plate counts of all the samples using 10-fold serial dilutions prepared in 0.1% peptone water.^3^ Simultaneously bacterial growth was monitored by measuring absorbance with a spectrophotometer (Nova Spec II, Pharmacia) at 600 nm.^10^ All the experiments were replicated twice.

**Antimicrobial assessment. **The inhibitory effect of *Lactobacillus* strains on selected clinical reference strains was determined by the well-diffusion method. For the agar well diffusion assay, an overnight culture of the indicator strain (*Escherichia coli, bacillus cereus, salmonella entritidis and Listeria monocytogenesis*) was used to inoculate to (Brain Heart Infusion) BHI agar growth media at 37 °C (approximately 10^6^ cells mL^− 1^ of indicator strains were overlaid onto BHI agar plates). Wells of 5mm diameter were cut into agar plates and 50 μL of *Lactobacillus* culture supernatant fluid that probably containing antibacterial activity was added to each well. Inhibitory zone of *lactobacillus* were checked after 24 hour incubation at 37 °C.^13^

**Statistical analysis. **The data were analyzed using Microsoft Office Excel 2007 and confirmed by Minitab 15 software. All values were stated as the mean ± SD at the *p* value < 0.05. 

## Results

All isolated *Lactobacillus* were taken from 24 hours cultured MRS broth (exponential growth phase) and directly subjected to each of the two stress factors: low pH, bile salts.

**Fig. 1:**
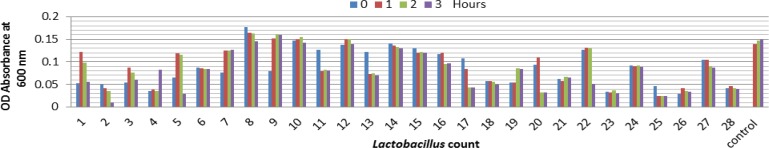
Tolerance of isolated *Lactobacillus* to pH=2.

**Fig. 2:**
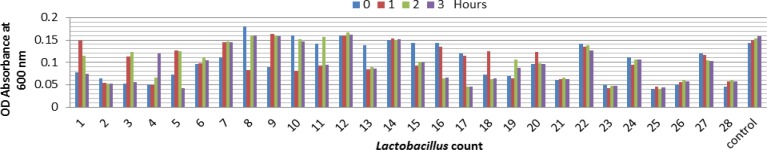
Tolerance of isolated *Lactobacillus* to pH = 3.

**Fig. 3:**
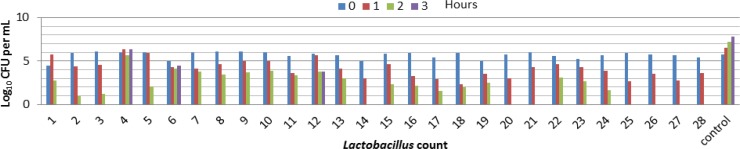
Isolated *Lactobacillus* growth and viability in pH = 2.

**Fig. 4:**
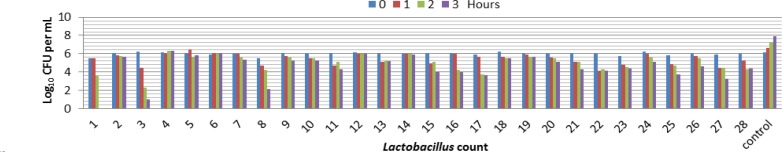
Isolated *Lactobacillus* growth and viability in pH=3.

**Tolerance to Acid of** ***Lactobacilli*** **. **The results of our study are shown in figures 1 and 2. The viability of *lactobacilli* decrease significantly after incubation at pH 3.0. The general comparison of all strains examined for significant differences in the cell viabilities after 1 and 3 hours of treatment indicated the similar pH tolerance (Figs.3 and 4). Below pH 3 the number of bacteria in the medium decreased because of the loss of viability. At pH ≤ 2.0 none viable bacterial cells were detected after the first hour suggesting most isolates were killed by severe pH.

**Antibacterial effect of** ***Lactobacilli*** **.** All isolated *lactobacillus* did not have inhibitory effect on *E. coli, Salmonella entritidis* and *Bacillus cereus*. Only one isolates of twenty eight lactobacillus, number four of isolates namely *Lactobacillus casei*, had inhibitory effect with 19 mm inhibitory zone on *Listeria monocytogenesis* after 24 hour incubation at 37 °C.

**Fig. 5:**
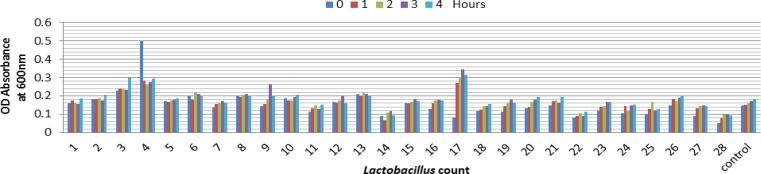
Tolerance of isolated *Lactobacillus* to 0.3% Bile concentration.

**Fig. 6:**
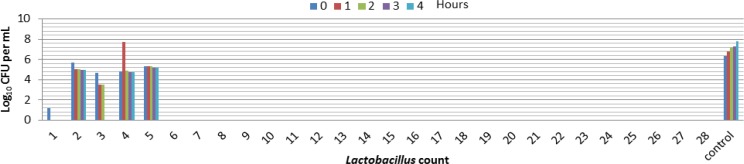
Isolated *Lactobacillus* growth and viability in 0.3 % Bile concentration.

**Tolerance to bile of** ***Lactobacilli*** **. **Bile salts present in the bacteria cultures were much more effctive on bacterial viability than effect of pH ≤ 3.0 (Figs. 5 and 6). The most resistant strain for o.3% bile concentration was samples number 2, 3, 4 , 5 that belongs to *Lactobacillus plantarum *( no. 2 and 3) and *Lactobacillus* casei( no. 4 and 5 ). It seems that number 2 , 4 and 5 bacteria were most resistant than other isolated bacteria to bile salts. Other strains could not survive at 0.3 % Bile concentration.

## Discussion

Chan* et al*. reported that acids such as the hydrochloric acid (HCl) found also in human stomach, disrupt the biomolecules of cells, such as fatty acids , proteins and DNA.^14^ Low pH environments can inhibit the metabolism and reduce the growth and viability of *Lactobacilli.* Other studies also confirmed that exposing to gastric acid with pH ≤ 2 after 3 hours incubation caused a reduction in the viability count of the bacteria intensively. ^14^^,^^15^ According to Prasad *et al.* and chan *et al*.*,* the threshold point to state acid resistance in this research was set at pH = 2 and pH = 3 for 3 hours incubation, as it simulates bacterial residency in the stomach.^14^^,^^16^ This is in accordance with findings from Liong and Shah which stated that resistance at pH = 3 was set as standards for acid tolerance of probiotic culture. Results in Figs 1- 4 indicate the strong inhibition on the viable bacteria numbers at pH = 2 and 3 (Figs. 3 and 4). In this study the bacterial growth decreased with increasing duration at pH = 2 and remained constant at higher pH (pH = 3). A Significant decrease in absorbance (OD) was not observed in all strains with decrease in pH (Figs. 1 and 2). It is probably because of the adaptation ability of lactobacillia to acid at the time of their presence in MRS broth.

During the evaluation of bile tolerance by growth studies, the growth abilities of the isolated strains in their culture media can be assessed. After bacterial exposure to bile salts, disruptions of cellular homeostasis occurred that caused the dissociation of lipid bilayer and integral protein of their cell membranes, resulting in bacterial content leakage and finally death of cell.^15^ All strains isolated in this study that could not tolerate the pH ≤ 2 also could not grow in the presence of bile salts except of isolates 2,3,4 and 5 that can survive at 0.3 % Bile concentration for 4 hours (Figs 5, 6). Several in vitro and in vivo experiments on antibacterial effect of different *Lactobacillus* on *Clostridium difficile*,* Campylobacter jejuni*, *Campylobacter jejuni*, *E. coli *have been performed. The isolates of this study have no active effect and the observed ability to inhibit the growth of *Escherichia coli*, *Bacillus cereus*, *salmonella entritidis* and *Listeria monocytogenesis* except one Isolate of *lactobacillia* that can inhibit growth of *Listeria monocytogenesis*. This isolate was *Lactobacillus casei* with number four in the numbered list. Other studies showed also that some strains of *Lactobacillus casei* had an inhibitory effect on different indicator bacteria.^17^^,^^18^ There are many strains among lactobacilli with documented probiotic ability, thus they have a more application in prevention of infection. Their inhibitory action is due to production of lactic acid, bacteriocins or H_2_O_2_ and deacetyl. The results obtained in this *in vitro *study may not completely reflect their performance *in situ *as many other physiological conditions that might affect the survival of the strains.

It should be emphasized that statistical analysis did not prove the significance of differences observed between the survival of *Lactobacillus* strains. Temperature during the fermentation process as well as during the inoculation period and most importantly during transportation can cause some strains low count.^19^

The protective effect of food matrix may also prevent the bacteria from bile exposure and hence, giving rise to the increased bile resistance of the strains.^20^

The results of this study showed that Koozeh cheese can be consumed as a native reserve for benefit bacteria such as probiotics, although extra studies should be done for determining the probiotic effects of isolated *lactobacilli* and other lactic acid bacteria of Koozeh cheese.
